# Mental Rotational Ability Is Correlated with Spatial but Not Verbal Working Memory Performance and P300 Amplitude in Males

**DOI:** 10.1371/journal.pone.0057390

**Published:** 2013-02-20

**Authors:** Gregory J. Christie, Charles M. Cook, Brian J. Ward, Matthew S. Tata, Janice Sutherland, Robert J. Sutherland, Deborah M. Saucier

**Affiliations:** 1 Department of Neuroscience, Canadian Centre for Behavioral Neuroscience, University of Lethbridge, Lethbridge, Alberta, Canada; 2 Department of Psychology, Simon Fraser University, Burnaby, British Columbia, Canada; 3 Faculty of Science, University of Ontario Institute of Technology, Oshawa, Ontario, Canada; University of Milan, Italy

## Abstract

This study investigated how both sex and individual differences in a mental rotation test (MRT) influence performance on working memory (WM). To identify the neural substrate supporting these differences, brain electrical activity was measured using the event-related potential technique. No significant sex differences were observed in a test of verbal WM, however males were significantly faster than females to respond to probe stimuli in a test of spatial WM. This difference was no longer significant after controlling for differences in MRT score, suggesting that rotational ability mediates performance in the spatial memory task for both sexes. A posterior P300 was observed in both tasks as participants encoded information into memory, however the amplitude of the P300 correlated with RT in the spatial task but not in the verbal task. Individual differences in the MRT also correlated with RT and with the amplitude of the P300, but again only in the spatial task. After splitting the analysis by sex, partial correlations controlling for MRT revealed that for males, individual differences in rotational ability completely mediated the correlation between the P300 and RT in the spatial task. This mediating effect was not observed for the female participants. The results therefore suggest a relatively stronger association in males between innate mental rotational ability, spatial memory performance, and brain electrophysiological processes supporting spatial memory.

## Introduction

Although there are no systematic differences in intelligence between adult males and females [1], certain tasks may elicit relatively differential performance including a male advantage for several tests of spatial reasoning and the manipulation of mental imagery [2]. For example, in the Vandenberg and Kuse mental rotations test [3] (MRT), participants must mentally rotate a series of probe items and assess which of them match a reference item [4]. The robust individual differences in the accuracy with which participants can perform this test correlate with other measures of spatial aptitude, including the speed that participants learn complex routes on a map [5] and the efficacy with which they learn to navigate a virtual maze [6]. The MRT also yields one of the largest [7], [8] cognitive sex differences between adult males and females, with a male advantage of approximately 0.8 – 1.0 *d* [9]–[13]. Furthermore, whereas female performance on other tests of spatial cognition (e.g. spatial visualization) have improved substantially through the period from 1945–1995 [7], the magnitude of the sex difference in the MRT has remained largely unchanged [14]. The male advantage for covert rotation is also evident for the rotation of simple two-dimensional shapes [15] and real-world objects such as animals, tools, and persons, although effect sizes are smaller [16].

To mentally rotate an object, participants most likely create a mental representation of the item and its component parts, maintain that representation, and continuously update the representation as the object rotates [3], [4]. These processes are consistent with the currently supported model of working memory (WM) [17], [18], in which a central executive can access and manipulate information retained in dissociable buffers for visuospatial and phonological information. Appropriately, performance on the MRT correlates primarily with spatial (and not with verbal) WM capacity [19], and individuals with greater WM capacity rotate objects with greater accuracy through a larger angle and across multiple axes [20].

Presumably then, the male advantage for the MRT should manifest as a male superiority in general tests of spatial WM. However, this in fact is not always observed. Unlike mental rotation, sex differences in spatial WM are less consistent and where observed exhibit moderate effect sizes [21]–[23]. For example, there is a moderate female advantage on some tests of object location memory (often tested by tasks like the children’s game, *Concentration*), in which the locations of numerous items in an array must be held in WM in order to locate matching symbols or shapes among the array. Females typically require fewer trials than males to complete the task [24], [25]. Further, females are often more accurate than males at identifying items that have switched positions with each other within a previously examined array [26]. However, others have reported a male advantage for tasks of spatial WM, including backward wayfinding test and on three different tests of object location memory [27], as well as a task that required participants to mentally trace a pattern through a memorized array of black and white tiles. Interestingly, no sex difference was observed in terms of the ability to accurately recall the positions of those tiles [28]. In another study, males outperformed females on a spatial variant of the classic digit span task, suggesting greater WM capacity in the male participants in that experiment [29].

A recent study conducted by Scott Kaufman sought to directly address the relationship between sex differences in spatial aptitude (including MRT) and sex differences in verbal and spatial WM [30]. Although no sex difference was observed in the test of verbal WM, a male advantage was observed on the MRT, on the space relations subtest of the differential aptitude test (DAT-sr), and on two tests of spatial WM in which participants had to sequentially memorize locations presented on a computer screen while also responding to task-irrelevant verbal information. Using structural equation modeling, the author demonstrated that although the male advantage for the DAT-sr was completely mediated by differences in spatial WM performance, the male advantage on the MRT was not. In other words, although the male advantage on the MRT arises partly because of differences in spatial WM performance, some other cognitive component also contributes towards the male advantage for this test.

Given the complex and often discrepant nature of these behavioral results, it should come as little surprise that there are relatively few functional neuroimaging studies into the neural origins of sex differences in WM. Most functional MRI studies have instead focused on sex differences in mental rotation, typically reporting that the male advantage correlates with enhanced activation of right-hemisphere posterior parietal cortex [31]–[33]. With respect to sex differences in spatial WM, there is evidence suggesting that the male advantage may reflect differential activation of the precuneus and areas of the frontal lobes [34], [35], and that males may preferentially engage right-hemisphere structures to facilitate spatial processing [36], [37].

The brain processes mediating WM may also be investigated using EEG and the time-locked event-related potential (ERP) technique, which affords excellent temporal resolution to investigate millisecond-level changes in brain electrical activity. The ERP approach has been used to contrast brain activity during: object and spatial WM [38]; verbal and spatial WM [39]; engagement of WM during mental rotation [40]; and to investigate the neural bases of individual differences in WM capacity [41]. Although several ERP components have been linked to the encoding, maintenance, and retrieval processes subserving WM, one of the most consistently observed effects is that of a positive-going deflection in the ERP over central-parietal scalp areas: the P300. The P300 is an endogenously generated neuroelectric response observed in a wide variety of cognitive tasks reflecting the informational content and task relevance of the stimulus, rather than the processing of the physical properties of the eliciting stimulus. In the context of WM, the P300 is usually observed during the initial presentation of the to-be-remembered stimuli and may reflect the activation of attentional and memory networks used to facilitate information processing [42]. Concordantly, individuals with greater WM capacity tend to exhibit a larger P300 as they encode information into WM [43], [44]. From this, it may be presumed that any advantage afforded by sex during WM should manifest as an enhanced P300 as participants encode stimuli. However, although some ERP studies have investigated the neuroelectric origins of sex and individual differences in mental rotation [45], [46], to our knowledge there exists no published study that has used the ERP approach in investigate sex differences in WM, nor has any ERP study investigated how mental rotational ability influences brain activity during WM.

A pilot study conducted by Hoesing and her colleagues [47] used a novel match-to-sample task to investigate sex differences in spatial and verbal WM. In three experiments, males were significantly faster than females at responding to probe stimuli in a spatial WM task in which participants had to memorize specific locations within a grid [48]. Notably, the same visual stimuli are used for both the spatial and the verbal tasks, and the two tasks differ only in the instructions given to participants. This makes this paradigm well suited to the ERP technique because it ensures that any differences in brain electrical activity originate because of the cognitive demands of the task rather than simple, but often unavoidable, sensory differences among tasks.

There were three objectives to the present study. First, we sought to replicate and confirm the previously observed male RT advantage in this particular spatial WM task. Second, we tested the hypothesis that individual differences in spatial ability, assessed via the MRT, is the factor mediating this sex difference in the spatial task. Finally, as the neural mechanisms supporting the male advantage are as yet unknown, we sought to identify the neuroelectric mechanisms by which performance differences arise in this spatial WM task.

## Materials and Methods

### Mental Rotations Test

The Vandenberg & Kuse [3] Mental Rotations Task (MRT) was used as a screening test to assess individual differences in mental rotation ability. In this task, participants had to choose two of four 3-dimensional figures that matched a reference figure. The four alternatives were rotated, thus to choose the correct figure the participant had to covertly rotate the target to determine a match. There were 12 items and participants had 4 minutes to complete the task. The total score was corrected for guessing, with one point for correct choices and a one-point penalty for incorrect choices (maximum score  =  24; minimum score  =  0).

### Participants

Participants were recruited in two phases. In the first phase, participants were selected from another study investigating the relation between personality variables and spatial ability [49]. The first study had no overlapping tasks with the current one, with the exception that participants performed the MRT in the first study rather than during this one. Of the 191 participants in the first study, we recruited 41 who performed at least one standard deviation better or worse than their sex’s mean score on the MRT. Two participants were rejected after testing due to behavior that was inconsistent with actual performance of the task (the mean accuracy of their responses was at chance) and one participant was rejected due to excessive noise in the recorded EEG. There were thus 10 high-ability (Mean MRT score  =  16.40) and 11 low-ability (*M*  =  3.45) females, and 8 high-ability (*M*  =  20.25) and 9 low-ability (*M*  =  7.33) male participants entered into the analyses from phase one. In the second phase, 24 random participants were recruited from the undergraduate student body at the University of Lethbridge. MRT ability was not controlled for in this phase. Two participants were excluded due to data corruption in the recorded EEG and there were thus 11 male and 11 female participants recruited in phase two. Collectively, 60 participants were entered into the behavioral and EEG analyses (mean age  =  23.78 years old; *SD*  =  7.41; 5 left handed). Sex and group differences were quantified using a 3 (Low, High, Random MRT)×2 (Sex) repeated-measures ANOVA, and all subgroups were significantly different from each other (see Results section). The participants were all undergraduate students at the University of Lethbridge and had normal or corrected to normal vision. Participants received compensation of either $20 CAD or 2% bonus course credit for their participation. The University of Lethbridge Human Subject Research Committee approved this study, and the research was conducted in accordance with the principles expressed in the Declaration of Helsinki.

### Spatial WM Task

**Figure 1:**
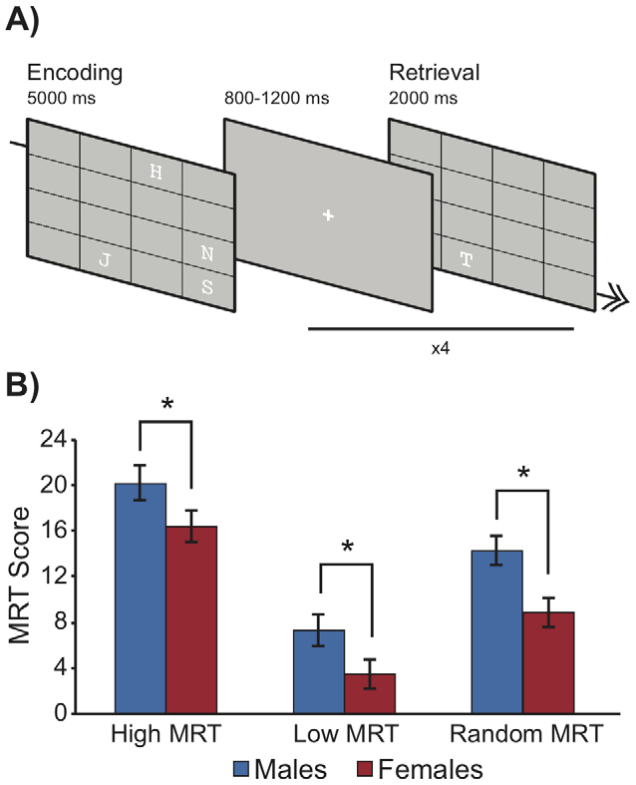
Task design and recruitment results. (A) Task design depicting a true probe in the spatial memory task. The same stimuli were used for the verbal memory task, but participants were instructed only to memorize the identity and not the locations of the letters in the grid. (B) Results from the Vandenberg and Kuse MRT. The maximum score was 24. Participants were recruited for this study who scored either very high or very low on the MRT, along with a number of randomly recruited participants (see Methods for details). Males outperformed females in all subgroups (see Results).

For this task (Figure 1), participants were seated comfortably in a normally lit room 180 cm from a 20” LCD computer monitor (Dell 2000FP). The forearm of the dominant hand rested on a table holding a standard computer keyboard. Stimuli were presented using the E-Prime program (Psychology Software Tools Inc, Pittsburgh PA) and consisted of English consonants measuring approximately 4 cm tall×2.5 cm wide. Letters were white and presented against a light gray background. The letters were displayed inside a 4×4 grid of 16 squares; the grid was dark gray and measured 30×30 cm. Testing began with the presentation of the initial array of 4 letters within the grid (encoding phase). Participants were given 5 s to remember only *the locations of the letters* and to ignore their identities. Following the 5 s encoding period, a fixation cross was displayed for a random duration of 800–1200 ms (rectangular distribution), and then the grid was again presented, this time displaying only a single probe letter (retrieval phase). Probes were always different than the studied items; that is, if the initial items studied were 'B', 'F', 'G' and 'Z', then the probe would be for e.g. 'Q'. For each array to be memorized there were four probe trials and each probe trial was presented for 2 s with a 1 s pause between each presentation. Within this 2 s window, participants were required to indicate whether or not the location had been occupied in the original grid by pressing a key on the keyboard. On average 50% of probes were true and 50% were false. Any particular block of trials may have had 0, 1, 2, 3, or 4 true probes, and the experiment was arranged such that participants could not guess what any one probe might be. Participants completed a total of 60 arrays and thus were presented with a total of 240 probe items. Participants were instructed to respond as quickly and as accurately as possible.

### Verbal WM Task

The parameters and equipment for this task were the same as in the spatial WM task (above) except that participants were instructed to remember only the *identities* of the letters and to ignore their locations during the encoding phase. As with the spatial WM task, probe items were always incongruent with the studied items; that is, the probe items were always in different locations than in the studied array.

### Procedure

Participants provided informed consent and then completed a demographic questionnaire followed by the Vandenberg and Kuse Mental Rotations task (n.b.: participants recruited in phase one completed this task in advance). Participants were seated in the experimental room and the electrode net placed on their head. Participants were then given a brief tutorial explaining both the spatial and verbal WM tasks. After the tutorial, one minute of baseline EEG was recorded (not analyzed) during which participants were instructed to relax and focus on a fixation cross displayed on the experimental computer. To control for order effects, half of all participants began with the spatial task and half began with the verbal task. Each task took approximately 20 minutes to complete with a break at the halfway point of each session, the length of which was determined by the participant. At the end of the first condition (verbal or spatial), the experimenter entered the room and refreshed the electrode net with the electrolyte solution (approximately 3 minutes) and then initiated a second one-minute baseline EEG recording before beginning the second condition. The average length of the experiment was approximately 47 minutes.

### EEG Collection

The EEG was recorded with a vertex reference at a 500 Hz sampling rate using 128 Ag-AgCl electrodes in a geodesic array (Electrical Geodesics Inc., Eugene, OR, USA). Impedances were maintained below 100 KΩ, an appropriate level for high input impedance amplifiers (input impedance  =  200 MΩ). The recorded EEG was highpass filtered at 0.01 Hz and lowpass filtered at 200 Hz. Electrode placement was recorded with a Polhemus Fast-Trak (Polhemus, Colchester, VT, USA) for later registration with the EEG dataset.

### Analysis

EEG data were imported into the BESA software package (Megis Software, Grafelfing, Germany) for further analysis. The record was visually inspected for bad channels and the signal from a small number of electrodes was replaced with interpolated signal (ocular, reference, and electrodes of interest were not corrected). The EEG was digitally re-referenced to an average reference (n.b. similar results were observed when using a virtual montage referenced to the average of the two earlobes [50]). Ocular artifacts were corrected using an adaptive artifact correction algorithm [51]; HEOG and VEOG threshold voltages were 150 µV and 250 µV respectively. Epochs with amplitude greater than 120 µV were rejected during automatic artifact scanning. The data were then lowpass filtered with a 20 Hz, 24 dB/octave zero-phase digital Butterworth filter. Trials with incorrect responses were excluded from subsequent analysis.

**Figure 2:**
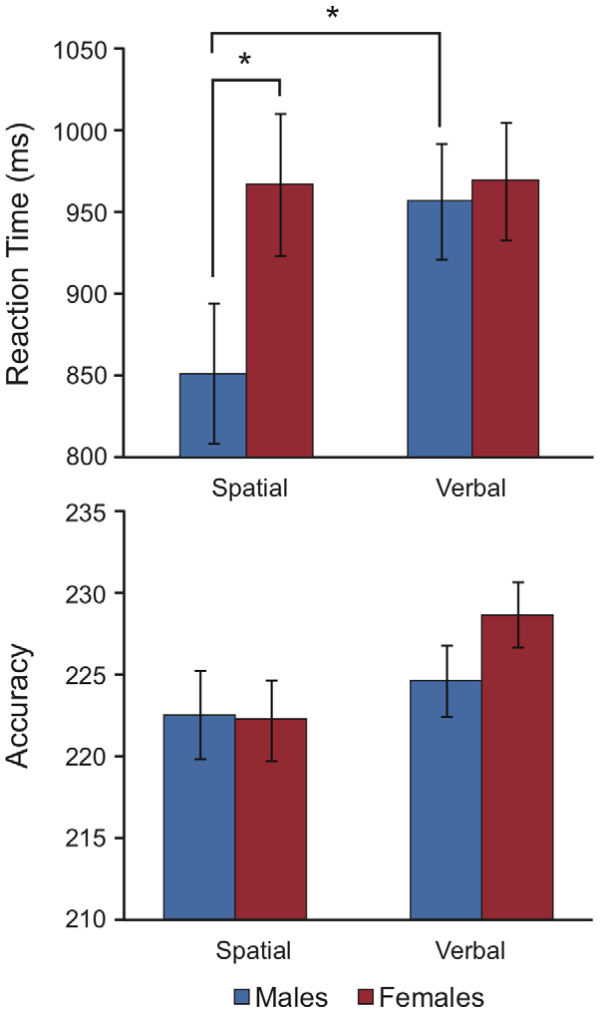
Behavioral results from the spatial and verbal WM tasks for the randomly recruited participants. Males were faster at the spatial task than females and were faster than their own performance on the verbal task. No significant differences in accuracy were observed.

Grand-averaged scalp ERPs were computed in a 1000 ms epoch with a 200 ms pre-stimulus baseline and an 800 ms post-stimulus period of interest for both the encoding and retrieval phases. For illustrative purposes, ERPs were also computed for the 14 highest- and 13 lowest-scoring MRT participants (quartile split). Visual comparison between the high- and low-MRT ERP waveforms did not reveal substantial differences during either of the two retrieval operations, but there were evident differences at posterior electrodes during the encoding operations for both WM tasks. ERPs were computed by averaging the activity at ten posterior electrode sites (denoted in Figure 2). Activity for the encoding operations was quantified by averaging the ERP waveform in the 400–500 ms period where the amplitude was largest.

To identify the relationship between MRT score and task performance, the analysis was expanded to include the entire sample (*N*  =  60) and three separate 2 (Sex) × 2 (Task) ANCOVAs were computed with RT, accuracy, and the amplitude of the encoding P300 as the dependent variables and MRT score as a covariate. Brain-behaviour relationships were assessed using the Pearson product moment computed between MRT score, RT, and accuracy for the spatial and verbal WM tasks, and the amplitude of the encoding P300 during the spatial and verbal WM tasks. Partial correlations controlling for individual differences in MRT score were also computed for both sexes and for males and females separately.

## Results

### Mental rotations task

A 3 (Low, Random, High MRT subgroup) × 2 (Sex) univariate ANOVA revealed a significant main effect of MRT category, *F*(2,54)  =  43.70, *p* < .001, and a significant main effect of sex, *F*(1,57)  =  15.92, *p* < .001. The Category × Sex interaction was not significant, *F*(2,54)  =  0.24, *p*  =  .78. Post-hoc comparisons (Bonferroni correction) revealed that males outperformed females in each of the three categories, and that each of the three categories was significantly different from the other, with MRT scores being highest in the High subgroup and lowest in the Low subgroup for each sex (Figure 1).

### Task Behavior

We first sought to replicate the male RT advantage in the spatial task reported in [48] by restricting the analysis to the randomly recruited participants. For RT, a 2 (Task) × 2 (Sex) mixed-model ANOVA revealed a significant main effect of task type, *F*(1,20)  =  6.01, *p*  =  .024, *η_p_^2^* **= ** .231; and a significant within-subjects Task × Sex interaction, *F*(1,20)  =  5.55, *p*  =  .029, *η_p_^2^*  =  .217. The between-subject main effect of Sex was not significant, *F*(1,20)  =  1.53, *p*  =  .231, *η_p_^2^*  =  .071. Post-hoc comparisons revealed that males (*M*  =  957 ms) were not significantly faster than females (*M*  =  969 ms) in the verbal task. However, males were significantly faster in the spatial task (*M*  =  852 ms) than their own performance in the verbal task and males were also faster than females in the spatial task (*M*  =  967 ms), albeit at a one-tailed level (*p*  =  .075).

**Figure 3:**
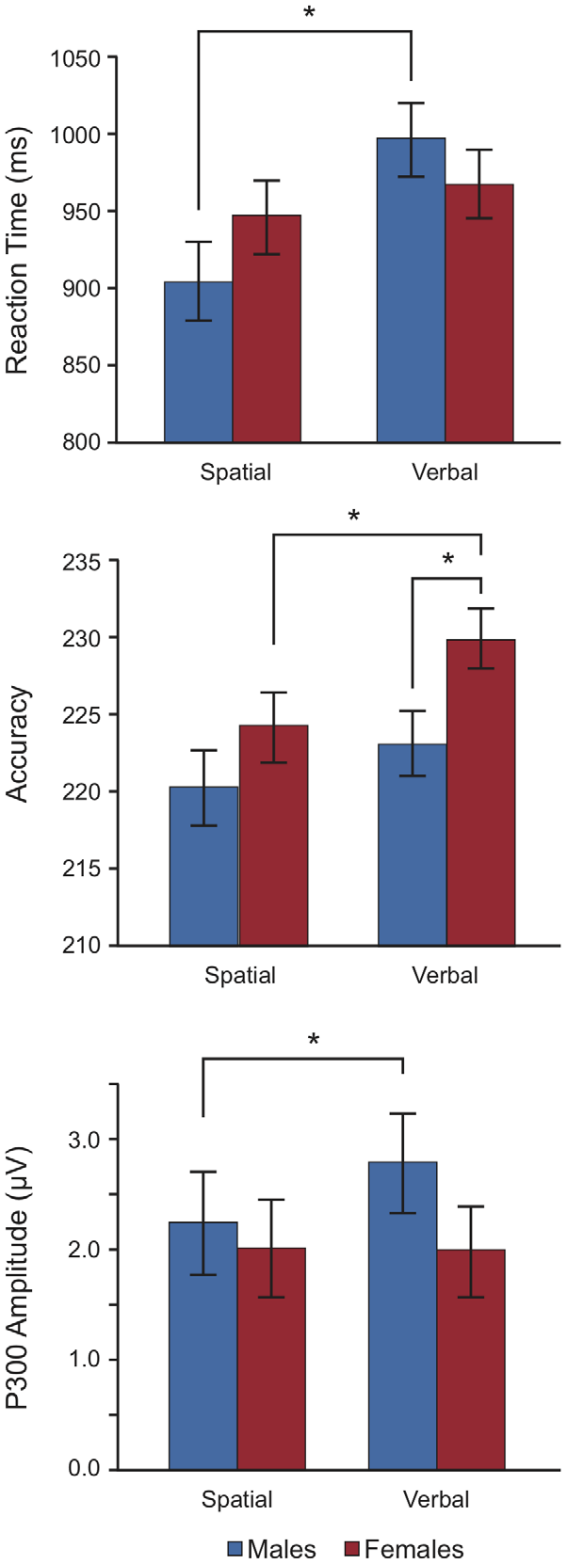
Behavioral and ERP results from the spatial and verbal WM test, computed for the entire sample at the median MRT  =  11.40. Although males were again faster at the spatial task than at the verbal task, there was no significant sex difference in RT in either task. Females were more accurate at the verbal task than males and were more accurate than their own performance at the spatial task. Males had a larger P300 during encoding in the verbal task than in the spatial task.

To determine how mental rotational ability influenced both task behavior and brain electrophysiology, the analysis was expanded to include the high- and low-scoring MRT participants recruited in phase 2. For RT, a 2 (Task) × 2 (Sex) mixed-model ANCOVA was performed with MRT score as a covariate. Within-subjects contrasts did not reveal a significant main effect on RT of task type, *F*(1,57)  =  1.73, *p*  =  .19, *η_p_^2^*  =  .029, nor was there a Task × MRT interaction on RT, *F*(1,57)  =  0.54, *p*  =  .47, *η_p_^2^*  =  .009. However, there was a significant within-subjects Task × Sex interaction, *F*(1,57)  =  5.44, *p*  =  .023, *η_p_^2^*  =  .087. MRT score was a significant between-subjects factor, *F*(1,57)  =  5.86, *p*  =  .019, *η_p_^2^*  =  .093; but sex was not, *F*(1,57)  =  0.05, *p*  =  .83, *η_p_^2^*  =  .001. Post hoc comparisons (Bonferroni correction) revealed that males were significantly faster in the spatial task (*M*  =  905 ms) than they were in the verbal task (*M*  =  996 ms), but that females were not significantly faster to perform the spatial task (*M*  =  947 ms) or the verbal task (*M*  =  967 ms), and there were no sex differences in speed for either the spatial or verbal tasks (Figure 3).

For accuracy, the 2 × 2 ANCOVA revealed a significant within-subjects main effect of task, *F*(1,57)  =  4.89, *p*  =  .031, *η_p_^2^*  =  .079, however neither the Task × MRT [*F*(1,57)  =  1.46, *p*  =  .23, *η_p_^2^*  =  .025] nor the Task × Sex [*F*(1,57)  =  0.58, *p*  =  .45, *η_p_^2^*  =  .010] interactions were significant. Both MRT [*F*(1,57)  =  18.16, *p* < .001, *η_p_^2^*  =  .242] and Sex [*F*(1,57)  =  4.26, *p*  =  .044, *η_p_^2^*  =  .070] were significant between-subjects factors. Post-hoc tests (Bonferroni correction) revealed that females were significantly more accurate in the verbal task (*M*  =  230) than were males (*M*  =  223), and were also significantly more accurate than their own performance in the spatial task (*M*  =  224); males were not any more accurate in the verbal task than they were in the spatial task (*M*  =  220). However, given that the maximum score for this task was 240 we suggest that we are approaching ceiling effects for both tasks (Figure 3).

Finally, to ensure that the male RT advantage in the spatial task was not the result of a speed-accuracy tradeoff, the Pearson product moment was computed between RT and accuracy for males and females for both the spatial and verbal tasks. A speed-accuracy tradeoff would be indicated by a positive correlation between RT and accuracy. Instead, RT and accuracy were negatively correlated for both males and females in both tasks (all *r* > -.425, all *p* < .025).

### EEG Results

Due to the large mismatch in trial numbers between the encoding (60 trials per subject) and retrieval operations (240 trials per subject), the EEG analyses were restricted to include only the first probe item in order to equate signal-to-noise. There were no significant differences in EEG measures when using this smaller subset of trials.

**Figure 4:**
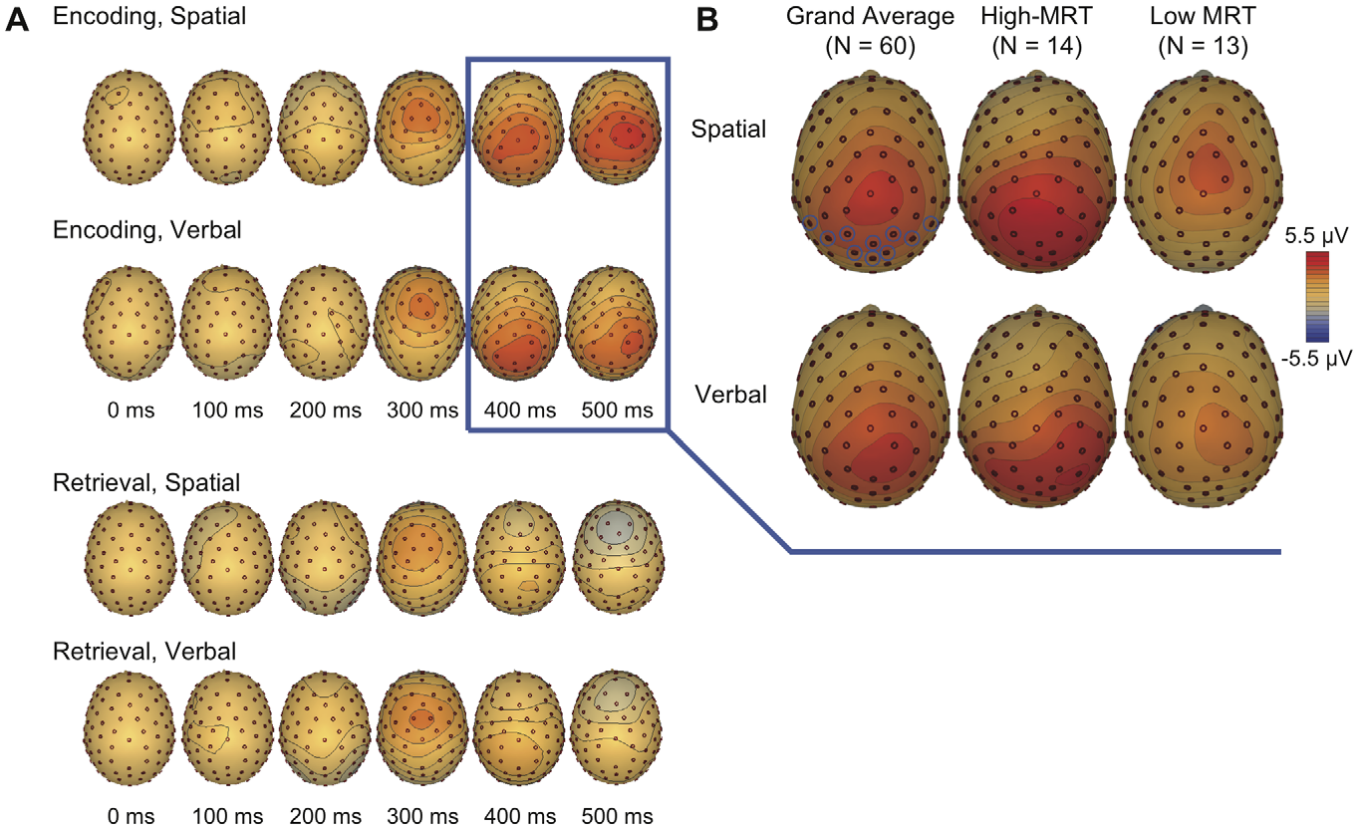
Topographic plots of scalp electrical activity. (A) Activity in the 0–500 ms period in 100 ms intervals for the encoding and retrieval processes for both WM tasks. (B) Scalp topographic plots of activity during the 400–500 ms epoch after the onset of the encoding array in the spatial (top row) and verbal tasks (bottom row). Grand-averaged scalp plots are depicted in the left column, along with representative plots for the 14 highest- (center column) and 13 lowest-scoring participants (right column) in the mental rotations task. ERPs were computed by averaging the EEG at ten posterior electrodes, denoted in blue.

Grand-averaged (*N*  =  60) scalp topographic plots for the encoding operation (400–500 ms post-stimulus) in the spatial and verbal tasks are depicted in Figure 4. Representative topographic plots are also presented for the 14-highest and 13-lowest scoring MRT participants. EEG values were averaged at ten posterior electrodes for subsequent analyses and are denoted in blue. There was a notable positivity over posterior scalp electrodes to the encoding phase and this positivity was greater and more posterior for high- relative to low-MRT participants.

**Figure 5:**
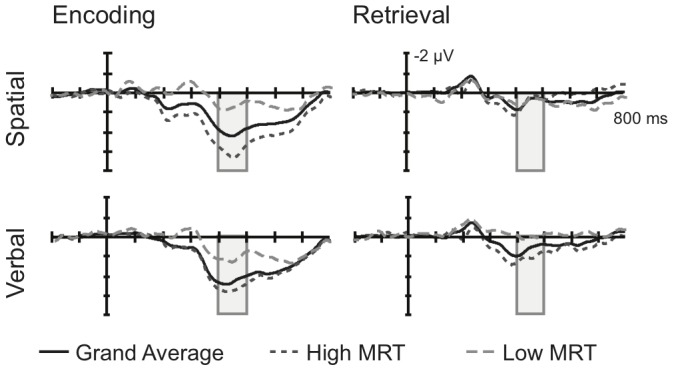
ERP waveforms to the encoding and retrieval operations, for the spatial WM task (top row) and the verbal WM task (bottom row). The grand-averaged ERP is depicted with a solid black line, high-ability participants with a dark grey dotted line, and low-ability participants with a light grey, dashed line. The 400–500 ms period was used for statistical analyses and is depicted by a grey box. Horizontal ticks represent 100 ms, vertical ticks represent 1 µV, and negative is plotted up.

ERP waveforms, averaged at the ten posterior electrodes, are presented in Figure 5 for the grand-averaged (*N*  =  60) dataset, and also for both the 14-highest (dark dashed line) and 13-lowest (light dashed line) scoring MRT participants. Activity during the retrieval phase was markedly smaller than during the encoding phase and did not obviously differ based on mental rotational ability. There was a notable P300 during the encoding phase for both tasks, and high-MRT participants had a notably larger posterior P300 than low-MRT participants for both the verbal and spatial tasks.

As with the RT analysis, we first investigated sex differences in the P300 in the randomly recruited participants. A 2 (Task) x 2 (Sex) ANOVA was performed with the amplitude of the P300 as the DV, however given the limited sample size (*N*  =  22), no significant within-subject main effects were observed for either task type [*F*(1,20)  =  2.58, *p*  =  .124] or for sex [*F*(1,20)  =  0.74, *p*  =  .40], nor was there a significant between-subjects main effect of sex [*F*(1,20)  =  1.86, *p*  =  .188].

We then expanded the analysis to include the high- and low-MRT participants and included MRT score as a covariate. The 2 × 2 ANCOVA for the activity elicited by the probe stimulus did not differ significantly based on Task, *F*(1,57)  =  0.95, *p*  =  .33, there was not a significant within-subjects Task × MRT [*F*(1,57)  =  2.69, *p*  =  .11] or Task × Sex [*F*(1,57)  =  0.04, *p*  =  .85] interaction, and the between-subjects effect of MRT score was not significant, *F*(1,57)  =  1.58, *p*  =  .22. However, the analysis of the P300 elicited by the memorization array now revealed a significant within-subjects main effect of task, *F*(1,57)  =  6.49, *p*  =  .014, *η_p_^2^*  =  .102, and a significant Task × MRT interaction, *F*(1,57)  =  4.41, *p*  =  .040, *η_p_^2^*  =  .072, however the Task × Sex interaction was not significant, *F*(1,57)  =  2.37, *p*  =  .13, *η_p_^2^*  =  .040. Neither MRT [*F*(1,57)  =  2.92, *p*  =  .093, *η_p_^2^*  =  .049] nor Sex [*F*(1,57)  =  0.70, *p*  =  .41, *η_p_^2^*  =  .012] were significant between-subjects factors. Post-hoc tests (Bonferroni correction) revealed that the amplitude of the P300 was significantly larger in males during the verbal (*M*  =  2.80 µV) than in the spatial task (*M*  =  2.24 µV); the amplitude of the P300 did not differ in females between the verbal (*M*  =  1.99 µV) and spatial tasks (*M*  =  2.01 µV).

### Brain-behavior correlations

Across both sexes, MRT score was significantly correlated with RT in the spatial task but not in the verbal task, and was correlated with the amplitude of the P300 in the spatial task but not in the verbal task. The amplitude of the P300 in the spatial task correlated with RT in the spatial task at the one-tailed level (*p*  =  .056), whereas the amplitude of the P300 in the verbal task was not correlated with RT in the verbal task (Table 1). After partialling out the shared variance accounted for by differences in MRT score, the weak correlation between the amplitude of the P300 and RT in the spatial task was no longer significant.

**Table 1:** Correlations Between Electrophysiology and Behavior.

		Encoding P300 Amplitude		Response Time	
	1 MROT Score	2 Spatial	3 Verbal	4 Spatial	5 Verbal
1					
2	.309*				
3	.195	.830** (.826**)			
4	–.358**	–.248 (–.155)	–.287* (–.237)		
5	–.227	–.138 (–.073)	–.093 (–.051)	.626** (.599**)	

Note: Partial correlations controlling for MRT score are presented in parentheses.

* p < .05 (± .254), 2-tailed

** p < .01 (± .330), 2-tailed

Because of the male advantage in the MRT, separate partial correlations controlling for MRT score were performed separately for males and females. For the males, controlling for MRT resulted in a failure of the amplitude of the encoding P300 to correlate with RT in the spatial task. For the females however, controlling for MRT now resulted in a significant correlation between the amplitude of the encoding P300 and RT for the spatial task (Table 2).

**Table 2:** Partial Correlations Between Electrophysiology and Behavior for Males and Females.

	Encoding P300 Amplitude		Response Time	
	1 Spatial	2 Verbal	3 Spatial	4 Verbal
1				
2	.819** (.852**)			
3	.092 (–.356*)	–.009 (–.357*)		
4	.014 (–.183)	–.040 (–.172)	.620** (.647**)	

Note: Females in parentheses.

* p < .05 (± .381 males; ±.355 females), 2-tailed

** p < .01 (± .487 males; ±.456 females), 2-tailed

## Discussion

The present work investigated how sex and individual differences in mental rotational ability influence performance on spatial and verbal WM. To elucidate the neural origins of these differences, scalp-recorded event-related potentials (ERPs) were obtained both when participants initially encoded information into WM and when they subsequently compared a probe item against the information retained in WM.

Consistent with previous findings, participants were faster but more error prone in the spatial task than in the verbal WM task [52]–[58]. When the analysis was restricted to the sample of randomly recruited participants (as opposed to participants who scored either very high or very low on a test of mental rotation; see below), we replicate the male advantage in the spatial task first reported in our pilot study: males were faster at the spatial task than their own performance on the verbal task, and they were faster than females at the spatial task. These results are also consistent with other studies that have demonstrated a male advantage on certain tests of visuospatial WM [27], [28], [30], [59], [60].

One of the main objectives of the present experiment was to investigate the reason why male participants are faster than females at performing this spatial memory task. We hypothesized that the RT advantage is associated with the same fundamental cognitive mechanism that gives rise to the male advantage in other tests of spatial aptitude, including mental rotation. We therefore obtained performance measures from all participants in the Vandenberg and Kuse mental rotations test (MRT), which is a known proxy for spatial aptitude and which also typically elicits stronger performance from males than from females. To broaden our sampling distribution, we also recruited volunteers from both sexes who scored either very high or very low on the MRT. Given that we made no attempt to equate MRT scores between the two sexes, males, unsurprisingly, outperformed females in each of these high-scoring, low-scoring, and randomly recruited subgroups. After expanding the behavioral analysis to include these additional participants, and when MRT score was entered into the analysis as a covariate, there was no longer a significant sex difference between males and females in the spatial memory task. A correlation analysis confirmed that the correlation between MRT score and RT was greater in the spatial task than in the verbal task. These results therefore support our theory that mental rotational ability is indeed the factor mediating behavioral performance on the spatial WM task.

However, after expanding the analysis and partialling out the variance accounted for by MRT score, two overall RT patterns remained constant: females were nevertheless equally fast at completing both WM tasks, and males were also nevertheless faster to respond to probes in the spatial task than they were in the verbal task. This is probably not the result of a speed-accuracy tradeoff engaged in by the male participants, as accuracy and speed were negatively correlated for both sexes in both tasks. A potential, albeit speculative explanation may be that female participants were more likely than males to process both the location and the identity of the stimuli in tandem, regardless of task type. Support for this theory comes from a study conducted by James and Kimura [61], which used a modified version of the object location memory paradigm described in Silverman & Eals [26]. In both studies, participants were shown an array of items and asked to memorize their locations. After a study period, these items were then replaced with an array of test items. In the original study performed by Silverman and Eals, some of the test items had *exchanged* places with each other. Participants had to indicate which of the items had swapped places, and females were significantly more accurate at this than were the males. In the study conducted by James and Kimura however, these items, rather than being in exchanged places, were instead *moved* to new, previously unoccupied positions. Females were just as accurate in this new version of the task, but the male accuracy decrement was completely recovered and they now responded as accurately as the females [61]. From this, the authors posited that females may be more likely than males to retain both the locations and the identities of items during memory tasks [2]. In the present study, participants were specifically instructed to remember only the identities of the letters (verbal task) or the locations of the letters (spatial task) within the array. However, we have no way of knowing the particular encoding strategy utilized by each participant. It may be the case that the female participants nevertheless retained both the locations and the identities of the letters during the encoding process, accessed either dataset with the same speed during the probe display, and thus took equally long to generate a response in both tasks.

The other central goal of this study was to identify *how* differences in WM performance – especially in the spatial task – are affected by differences in neural activity. For example, it was previously unknown if participants who responded faster in the spatial task did so because they more efficiently encoded information into WM, because they were better able to retrieve that information back from WM, or both.

The electrophysiological activity elicited by the probe stimulus was negligible and did not differ between the two tasks. This observation is consistent with other studies wherein the majority of the observed ERP differences during WM occurred during the encoding and the active maintenance of the stimuli, and not during their retrieval per se [38], [62]. By comparison, the presentation of the memorization array elicited a robust P300 in both the spatial and the verbal tasks as participants transferred the information to memory. From this, we infer that the initial process of encoding information into WM is a more cognitively demanding process than is the subsequent retrieval of that information from WM.

After correcting for differences in MRT score, in the female participants the amplitude of the P300 did not differ significantly between the two tasks. This electrophysiological result is homologous to the RT results, which also did not differ significantly between tasks, and further suggests that females may have retained both the spatial and featural properties of the memorized items in both tasks. In the male participants on the other hand, the amplitude of the P300 was larger in the verbal task than in the spatial task. This indirectly replicates other studies that have investigated electrophysiological differences between spatial and verbal WM (but not sex differences), and which have reported a larger P300 to the memorization of verbal stimuli [38], [57], [62]. During verbal WM tasks, a larger P300 is also elicited when participants use a rote-rehearsal strategy to memorize stimuli; the use of other, more elaborate encoding strategies elicits a smaller P300 [63]. Part of the difference in P300 in the current study may therefore be the result of a rote rehearsal strategy used by males in the verbal task but not in the spatial task. Further research is warranted.

In the present study, three striking brain-behavior correlations were observed: (i) MRT score was more strongly correlated with performance in the spatial task than in the verbal task; (ii) the amplitude of the P300 in the spatial task, but not in the verbal task, correlated positively with MRT score, being larger in amplitude in the high-MRT participants and smaller in the low-MRT participants; (iii) the amplitude of the P300 correlated with RT in the spatial task, such that the fastest-responding participants had a larger P300. This correlation was again not observed in the verbal task. Partial correlations controlling for MRT were performed in order to determine if MRT score was the factor mediating the brain-behavior correlation in the spatial task. This was indeed the case: the correlation between the amplitude of the P300 and RT was now no longer significant after factoring out the shared variance accounted for by MRT. However, it again appears that this effect was driven primarily by the male subjects, as splitting the partial correlation analysis by sex now revealed a significant correlation between the amplitude of the encoding P300 and RT in the spatial task in females (*r*  =  -.36) but not in males (*r*  =  .09). Collectively, this suggests that in males, mental rotational ability completely mediates both task performance during visuospatial WM and commensurate electrophysiological activity related to the encoding of spatial information. That an independent test of spatial aptitude so profoundly influences RT and brain activity during spatial WM has not previously been reported.

Although ours is the first study to investigate the three-way relationship between MRT, WM and brain electrophysiology, other studies have directly investigated the relationship between the P300 and mental rotation. It is known that as the angle of rotation increases, so too does difficulty and the length of time required to mentally rotate the object [4]. The amplitude of the P300 also scales linearly with the angle of rotation, decreasing in amplitude as the angle increases [64]. In other words, the P300 is seemingly a direct psychophysiological marker of mental rotation [65], [66]. Thus, it is very likely that some of the neural areas responsible for generating the P300 are also recruited while performing mental rotation. Our results now suggest that these areas are also recruited during spatial WM.

Unfortunately, the limited spatial resolution of the EEG technique typically precludes the source localization of the P300, thus complicating the question of *where* in the brain this activity originates. However, there is converging evidence from both electrophysiological recordings in primates and from human lesion studies that the electric generators of the posterior P300 exist in areas of temporal and parietal cortex [67]–[70]. Functional imaging studies have revealed that mentally rotating an object activates similar areas of posterior parietal cortex [71]–[73]. From this, we believe that the RT advantage in the spatial task afforded by high MRT score represents the enhanced recruitment of temporal and parietal structures to support the encoding of spatial information into WM. However, support for this theory awaits converging evidence from studies using more spatially precise imaging techniques.

Finally, we turn to sex differences in the MRT and the residual sex difference observed in the spatial task in the present study. We had hypothesized at the outset that the male RT advantage at the spatial task was linked to the more general male advantage for spatial processing, and that females should perform as well as males after correcting for differences in this ability. This hypothesis is only partially supported by the observed data. Although MRT score correlated with both behavioral performance and with electrophysiological activity during the spatial memory task, the results suggest that mental rotational ability is more innately linked to these measures in the male participants than in females.

Although sex differences in the MRT have been known for over thirty years, there remains considerable debate regarding the extent to which biological [2], [74] and environmental [75], [76] factors influence this difference. Superficially, the male advantage in the MRT seems to be nearly universal: it has been observed in multiple cultures throughout the world [77], [78] and can be observed in children as young as five using an age-appropriate test of mental rotation [79]. However, there is also evidence to suggest that the male advantage on the MRT is nuanced. For example, one recent study has reported no significant sex differences on a variant of the MRT that, like the Vandenberg & Kuse MRT, consisted of perspective drawings of three-dimensional shapes [80]. It also appears that part of the male advantage in the MRT originates perhaps not due to rotational speed but because females are slower to transform the two-dimensional depictions of the stimuli into three-dimensional mental representations. When the to-be-rotated stimuli are presented not as two-dimensional cartoons but as actual three-dimensional forms, females are significantly more accurate on the MRT [81], [82]. Finally, although performance on the MRT improves for both sexes after playing first-person-shooter style video games, the magnitude of the female improvement is far larger than is the male improvement [83]. This suggests that the female performance decrement in the MRT is recoverable with training.

There is also evidence suggesting that participants can perform mental rotation using one of two voluntary strategies, one visual and one motor [84], [85]. In the visual strategy, participants imagine the object rotating as if in response to an unseen exogenous force, whereas in the motor strategy, they envision grasping and applying an endogenous force to rotate the item. There are no apparent performance advantages to employing one strategy over the other [85]. However, both of these studies exclusively used male participants, precluding the possibility of determining if one sex preferentially utilizes one strategy over the other. It also remains unclear if the particular strategy used to complete mental rotation also affects performance during spatial WM.

In conclusion, our study suggests several interesting interpretations about sex and ability differences in spatial cognition. Males were faster on a test of spatial WM than females, but this advantage was predicated on individual differences in mental rotational ability. After correcting for differences in the MRT, this sex difference was no longer significant. A posterior P300 was observed as participants memorized information in both tasks, however the amplitude of this P300 correlated with MRT and with RT in the spatial task but not in the verbal task. In males, individual differences in MRT ability mediated the correlation between the amplitude of the P300 and RT, but in the female participants, partialling out the variance accounted for by MRT skill actually improved the correlation between the amplitude of the P300 and RT. This suggests a relatively stronger association between innate mental rotational ability and spatial memory in males. Future studies should consider that despite analogous P300 responses between men and women for spatial WM tasks, response latency differences in females might not reflect individual differences in spatial ability (as measured by MRT), but rather a different means of encoding spatial information during WM.
